# Yield, Grain Quality, and Starch Physicochemical Properties of 2 Elite Thai Rice Cultivars Grown under Varying Production Systems and Soil Characteristics

**DOI:** 10.3390/foods10112601

**Published:** 2021-10-27

**Authors:** Wichian Sangwongchai, Kanitha Tananuwong, Kuakarun Krusong, Maysaya Thitisaksakul

**Affiliations:** 1 Biological Science Program, Faculty of Science, Khon Kaen University, Khon Kaen 40002, Thailand; wichian_s@kkumail.com; 2 Department of Food Technology, Faculty of Science, Chulalongkorn University, Bangkok 10330, Thailand; kanitha.T@chula.ac.th; 3 Structural and Computational Biology Research Unit, Department of Biochemistry, Faculty of Science, Chulalongkorn University, Bangkok 10330, Thailand; kuakarun.K@chula.ac.th; 4 Department of Biochemistry, Faculty of Science, Khon Kaen University, Khon Kaen 40002, Thailand; 5 Salt-Tolerant Rice Research Group, Khon Kaen University, Khon Kaen 40002, Thailand

**Keywords:** rice (*Oryza sativa* L.), production system, soil characteristic, grain quality, starch physicochemical properties

## Abstract

Rice production systems and soil characteristics play a crucial role in determining its yield and grain quality. Two elite Thai rice cultivars, namely, KDML105 and RD6, were cultivated in two production systems with distinct soil characteristics, including net-house pot production and open-field production. Under open-field system, KDML105 and RD6 had greater panicle number, total grain weight, 100-grain weight, grain size, and dimension than those grown in the net-house. The amounts of reducing sugar and long amylopectin branch chains (DP 25–36) of the RD6 grains along with the amounts of long branch chains (DP 25–36 and DP ≥ 37), C-type starch granules, and average chain length of the KDML105 were substantially enhanced by the open-field cultivation. Contrastingly, the relative crystallinity of RD6 starch and the amounts of short branch chains (DP 6–12 and DP 13–24), B- and A-type granules, and median granule size of KDML105 starch were significantly suppressed. Consequently, the open-field-grown RD6 starch displayed significant changes in its gelatinization and retrogradation properties, whereas, certain retrogradation parameters and peak viscosity (PV) of KDML105 starches were differentially affected by the distinct cultivating conditions. This study demonstrated the influences of production systems and soil characteristics on the physicochemical properties of rice starches.

## 1. Introduction

Rice (*Oryza sativa* L.) is one of the most essential staple crops globally [1]. It is also among the most popular cultivated crops in almost every landmass around the world [2,3]. World rice production is currently more than 504.17 million tons between 2020 and 2021 [4], and it provides over 20% of caloric intake per day for more than 3.5 billion of the world population [5]. As the main rice-cultivating continent, Asia’s rice production and consumption accounts for approximately 90% of the rice production and consumption worldwide [6]. In Thailand, rice is the major food for 69.95 million individuals, and approximately 25.31 million tons and 7.50 million tons of milled rice were produced and exported in 2020, respectively [7]. This renders Thailand the world’s second-biggest exporter of rice, providing the country with approximately 7.44 billion US dollars [7]. The diversity in grain functionality of Thai commercial rice cultivars, which are not only consumed as cooked rice but are also used as an ingredient for starch-rich food and other biomaterial industries (e.g., noodle, confectionery, bakery, beverage, pharmaceutical and supplement, cosmetics, and animal feed), plays an important role in determining its market values [8,9,10]. The diverse cooking and functional properties of Thai commercial rice could meet the taste preferences and requirements of rice consumers that vary from one country to the next [11,12]. The Thai commercial rice cultivars that are mostly exported to foreign markets, are the high quality, long-grain, aromatic Jasmine rice (i.e., Khao Dowk Mali 105 (KDML105) and Kor Kho 15 (RD15)) and glutinous rice (i.e., RD6) cultivars [12,13,14]. When cooked, these rice cultivars provide superior cooking quality (e.g., optimum cooking time), natural aroma and taste, and good sensory attributes (e.g., softness), which lead to a consumer’s repeated buying behavior and a higher price range [12,14,15]. The unique starch physical and chemical characteristics (i.e., water solubility, swelling power, gelatinization, retrogradation, and pasting properties) determine the rice grain quality [16,17]. They are, in turn, intrinsically controlled by the starch chemical compositions and structural features including amylose content, chain length distribution (CLD) of the amylopectin branches, degree of polymerization, granule size, and protein and lipid contents [16,17,18,19]. Thai elite rice starch, especially RD6 and KDML105 starches containing low amylose content and proportion of long chain amylopectin, tended to display low gelatinization temperatures of starch, low setback (SB) and final viscosity (FV) values of starch gel, soft texture after cooking, and optimum cooking time [9,10,16,20].

The Thai elite rice cultivars, including RD6 and KDML105, are popularly cultivated in the Northeast Region of Thailand with the constantly increasing cultivated area of 2.58 and 4.47 million ha in the 2019/2020 growing season [14,21]. The sticky RD6 rice provides a long grain with good aroma, high yield, and resistance to diseases and insects; while the KDML105 cultivar is the long-grain rice with low level of amylose at 12–16%, which upon cooking provides soft texture with a good aroma [14]. Nonetheless, these elite rice cultivars grown in distinct cultivation locations display significant variations in grain qualities such as grain appearance (e.g., grain length or grain size and grain length-to-width ratio or grain shape), grain storage product composition, and starch physicochemical properties [22,23]. These grain quality parameters could be directly affected by the soil’s physical and chemical characteristics, which vary based on production systems and climatic conditions within each cultivation location [22,24,25,26]. In several agricultural areas, various types of crop production system are crucial agricultural practices that influence soil physical characteristics and play important roles in maintaining good soil condition [23,26,27]. Good soil condition includes the availability and movement of water and air in the soil as well as the existences of organic matter (OM), lime requirement (LR), and three major elements (i.e., nitrogen (N), phosphorous (P), and potassium (K)) throughout the plant growth stages [26,28,29,30]. These are greatly necessary for growth and development of the rice plant, and eventually improve its yield and grain eating and cooking quality [23,26,28,31]. Not only the nutrient deficiency condition but also the nutrient toxicity condition owing to excessive supplies of nutrients can enhance the invasion of pathogen and insects [32,33]. The deficiency of micronutrients (e.g., iron (Fe)) also affected numerous metabolic processes such as DNA synthesis, respiration, and photosynthesis [34,35]. As a result, the rice plant exposed to nutrient imbalance typically shows characteristic symptoms, such as poor root formation, thinner stems, folded leaves, pale green and dark brown necrotic spots, and yellow, burnt-orange and dying leaves, which ultimately lead to the retardation of normal growth and the decreases of tillering, panicle number, and yield ability of the rice [32,33,34,35,36,37]. For instance, nitrogen and phosphorous deficiency in soil can cause the decreases of rice photosynthesis, growth, development, yield, and grain storage product compositions (e.g., amylose and protein contents) [30,37,38,39]. Furthermore, soil potassium deficiency is a main cause of declines in translocation of photosynthetic products and other metabolites from leaves to pollen grains at the reproductive stage, resulting in spikelet sterility, unfilled grain percentage per panicle, reduced grain size and grain weight, and yield loss in rice [31,40]. In addition, excessive application of fertilizer to the soil results in nutrient imbalance in the plant cytosol, which leads to nutrient losses, and causes damage to the rice plants via loss of ion homeostasis [36,41]. Furthermore, the ion imbalance could interfere with the genes encoding the starch synthases at the transcriptional levels during the grain filling stages of rice [42]. Together, delayed seed development and maturation was observed, rendering the decline in rice yield and grain quality [43]. Additionally, high-level N and K applications induced the increment and diminution of protein and amylose contents in the rice grain, respectively, which deteriorated the eating quality of cooked rice by hardening the cooked rice texture [31,44,45,46,47].

Although a number of studies have focused on the effects of soil’s physical and chemical characteristics on rice yield and grain quality, no holistic data regarding the influences of production systems and soil characteristics on rice yield, grain storage biomolecule composition (i.e., reducing sugar, starch, nitrogen (N), crude protein, and amylose contents), starch molecular structure (i.e., granular size, relative crystallinity, degree of polymerization, and chain-length distribution of amylopectin branches), and starch physicochemical properties (i.e., swelling power, solubility, and thermal and pasting properties) have been provided. The objective of this study was thus to unravel whether the different production systems and soil characteristics could directly affect the rice productivity, grain quality, and ultimately the starch physicochemical properties of two elite Thai rice cultivars, namely, the Jasmine rice (KDML105) and the sticky rice (RD6) cultivars. Our results will establish the link between rice production systems with differing soil characteristics and their influences on yield, grain quality, and the rice grain and rice starch applications in various starchy food industries, as well as pinpoint the effective practices to improve yield and grain quality of the two elite Thai rice cultivars.

## 2. Materials and Methods

### 2.1. Plant Materials and Growth Conditions

The two Thai rice (*Oryza sativa* L.) cultivars including RD6 and KDML105 were cultivated during the 2020 crop season from 11th July to 30th November 2020 in two cultivating locations. The experiment was divided into two groups. (i) The two tested rice cultivars were cultivated in 8” plastic pots containing commercial potting soil (DIN-WIEANG-KOW, Khon Kaen, Thailand) (one plant per pot) placed in a net house at the Faculty of Science, Khon Kaen University in Khon Kaen province, Thailand, and was called ‘net-house pot production system’. (ii) The two rice cultivars were planted in the fields of Khao Wong district in Kalasin province, Thailand, and was called ‘open-field production system’. Nine replications per cultivar for net-house pot production and open-field production were evaluated in a Randomized Complete Block Design (RCBD). In addition, Thermo-Hygrometers (Deltatrak, Pleasanton, CA, USA) were installed to monitor environmental temperature and relative humidity at 9:00 a.m. on alternate days throughout growth stages of rice plants. The average temperature and humidity were 28.73 °C and 72.32%, respectively, during the 2020 crop season (Figure S1). The soils were collected from three different sources including commercial potting soil mixture, RD6 field soil, and KDML105 field soil to analyze the physical characteristics including soil textural classes (i.e., sand, silt, and clay), and chemical characteristics including organic matter (OM), lime requirement in terms of pure CaCO_3_ (LR), total nitrogen (N), available phosphorus (P), available potassium (K), cations exchange capacity (CEC), water pH, and electrical conductivity (EC) at the Center of Agricultural Commodity Standards Certification, Faculty of Agriculture, Khon Kaen University. The results are displayed in Table 1.

### 2.2. Plant Productivity and Grain Morphology

Rice productivity including the number of panicles per plant, total grain weight per plant, 100-grain weight, and the percentage of grain fertility (i.e., the number of fully filled grains per 100 grains) were recorded. To examine grain appearance, approximately 30 grains were randomly placed on black paper, and then an image of the rice grains was taken. Grain morphology parameters of 10 grains per plant, including grain length and width, were measured using Vernier calipers (ABLETOOL, Bangkok, Thailand). Then, the width and length were used to calculate grain width-to-length ratio, perimeter, and volume [48,49].

### 2.3. Storage Product Composition

Mature caryopses at the complete panicle maturity stage [50] were collected, air-dried, and stored as paddy at 4 °C in the sealed polyethylene bags until analysis. Reducing sugar and starch contents of the rice flour was measured according to previously described methods [51] with slight modifications. The milled rice was thoroughly homogenized to a fine powder using a manual coffee grinder (CG-01, Zhejiang, China), and the rice flour powder was sieved through a 35 mesh (0.50 mm) sieve (Humboldt, Raleigh, NC, USA) [52]. Grain reducing sugars were extracted by boiling the suspension of 200 mg rice flour powder in 2 mL of 80% (*v/v*) ethanol three times. Then, 300 μL of the ethanol-soluble supernatant containing reducing sugar was dried using Speed Vac (LaboGene, Kolkata, India), and the residue was then reconstituted in 100 μL double-distilled water and kept at 4 °C for sugar assay. Total starch content was measured from the ethanol-insoluble pellet by digesting to glucose [51]. Finally, the quantity of reducing sugars in the reconstituted sugar extracts and the glucose digested from starch fractions were analyzed using 3,5-dinitrosalicylic acid method with glucose as standards [48]. Protein content of the rice flour was quantified as total nitrogen content using the AOAC Kjeldahl method. Then, a factor of 5.95 was applied to convert nitrogen (N) values to crude protein content (N × 5.95). Percentage of amylose content of the rice starch was determined by a concanavalin A method following the Megazyme Amylose/Amylopectin Assay Kit manufacturer’s recommendations (Megazyme, Wicklow, Ireland). The moisture content (%) of the starch powder was also assessed according to the standard AOAC method [9].

### 2.4. Starch Isolation 

Rice starch was isolated from the sieved rice flour powder [52] using alkaline extraction [53]. Filtered rice flour (10 g) was steeped in 50 mL of 0.2% (*w/v*) sodium hydroxide at 25 °C for 3 h to completely remove grain storage protein. The suspension was stepwise filtrated through 4 layers of cheesecloth and centrifuged at 4000× *g* for 20 min. The pellet was washed twice with double-distilled water, and then centrifuged at 4500× *g* for 20 min. The top layer of proteins was removed, and the bottom starch layer was completely resuspended in 50 mL double-distilled water and neutralized by 1 M hydrochloric acid, and then centrifuged at 4500× *g* for 20 min. The starch was then washed twice with double-distilled water. To remove grain lipid, the starch was washed with 10 mL toluene and centrifuged at 4500× *g* for 20 min, and then washed with double-distilled water to completely remove the toluene residues. The white starch pellet was dried in a hot air oven (Binder, Tuttlingen, Germany) at 40 °C for 48 h [53], and ground to pass through a 35 mesh (0.50 mm) sieve (Humboldt, Raleigh, NC, USA) [52]. 

### 2.5. Starch Molecular Structure and Physicochemical Property

#### 2.5.1. Amylopectin Branch Chain Length Distribution

Isoamylase debranching of amylopectin accompanied by measurement of amylopectin branch chain length distribution (CLD) with a high-performance anion-exchange chromatography system equipped with a pulsed amperometric detector (HPAEC-PAD) was performed according to the method modified from Tangsrianugul et al. [19]. Each rice starch sample (50 mg) was dispersed in 3.8 mL of double-distilled water and then heated at 100 °C for 1 h. After cooling, 1 mL of the enzyme cocktail (3.6 U of pullulanase (Megazyme International Ireland Ltd., Wicklow, Ireland) and 0.5 U of isoamylase (Megazyme International Ireland Ltd., Wicklow, Ireland) in 100 mM, sodium acetate buffer pH 5.0) was added to the starch solution and incubated at 40 °C for 48 h. The debranched samples were added with 0.2 mL of 100 mM sodium chloride and 5 mL of deionized water, respectively, to inactivate the enzymes. After centrifugation at 5000 g for 5 min at 25 °C, the samples were filtered through 0.45 μm nylon filter. Then, 1 mg/mL of the filtrates were injected into the HPAEC–PAD system to determine the amylopectin CLD following the procedure modified from Kittisuban et al. [54]. The mobile phases included eluent A (150 mM NaOH) and eluent B (600 mM sodium acetate in 150 mM NaOH) and the flow rate was 1.0 mL/min. The debranched amylopectin linear branch chains were separated on a CarboPac PA-100 pellicular anion-exchange column (Dionex, Sunnyvale, CA, USA) with gradient elution of eluent B (10–40% for 0–30 min, 40–100% for 30–31 min, 100% for 31–34 min, and 100–0% for 34–35 min) at a column temperature of 26 °C and a flow rate of 1.0 mL/min. Finally, chromatographic separation of the amylopectin linear branch chains and maltoheptaose standard (Hayashibara Biochemical Laboratories, Okayama, Japan) were used to characterize amylopectin CLD with degree of polymerization (DP) up to 56. The average chain length (CL) of the sample was computed using the equipment software [9,54,55].

#### 2.5.2. Starch Granule Size Distribution

Starch granule size distribution of the isolated starch was analyzed by a laser diffraction particle size analyzer (Mastersizer 2000, Malvern Instruments, Malvern, Worcestershire, England). The 30 mg of isolated starch were dispersed in 1mL of 1% (*w/v*) sodium dodecyl sulfate. The starch suspension was used for size analysis at a pump speed of 2500 rpm. The results were reported as the size distribution in terms of the percent volumes (%volume) of equivalent spheres, and the starch granule size was expressed as median particle diameter (d(0.5)), which is the granule size at which 50% of all the granules by volume are smaller [56,57].

#### 2.5.3. X-ray Diffraction and Relative Crystallinity

X-ray diffraction patterns of rice starch powder were analyzed using X-ray diffractometer (Malvern Panalytical Empyrean, Taguig, The Philippines). Each rice starch sample was packed into a sample holder, and was scanned with an X-ray at a speed of 0.02°/s. The radiation was generated at a voltage of 45 kV and 40 mA with the scanning regions of the diffraction angle (2θ) ranging from 5° to 40°. The percentage of starch relative crystallinity was calculated following the formula [58].

Relative crystallinity (%) = Ac/(Ac + Aa)(1)

where Ac is the crystallized area on the X-ray diffractogram, and Aa is the amorphous area on the X-ray diffractogram.

#### 2.5.4. Swelling Power and Water Solubility

Swelling power (SP) and solubility (S) of rice starch were determined following the modified procedures of Vandeputte et al. and Wang et al. [59,60]. Briefly, 20 mg of starch powder were weighted directly into 2 mL screw-cap test tubes and mixed with 1 mL of double-distilled water. The starch suspension was then boiled in a water bath at 90 °C for 30 min with minimum shear condition. After cooling to room temperature, the suspension was centrifuged at 3000× *g* for 30 min. The supernatant was removed and transferred to a petri dish and baked in a hot air oven (Binder, Tuttlingen, Germany) at 105 °C to constant weight. The dried supernatant was then weighed to determine the rice starch solubility. Thereafter, the wet sediment was weighed for swelling power determination. The SP (g/g on a dry weight basis) and S (%) values were calculated using the following formulas:

SP = [wet sediment weight × 100]/[(dry mass of whole starch sample) × (100% − % of dry mass of total soluble molecules in supernatant)](2)

S (%) = (dry mass of total soluble molecules in supernatant × 100)/dry mass of whole starch sample(3)

#### 2.5.5. Starch Gelatinization and Retrogradation Properties

The sample preparation prior to analyses of gelatinization and retrogradation characteristics was carried out following the procedure modified from Tananuwong and Malila [61]. Starch powder of 3 mg was weighed into an aluminum pan (PerkinElmer kit no. 02190062, Waltham, MA, USA), and 9 μL of deionized water was added to achieve the starch to water ratio of 1:3. The sample pan was then hermetically sealed and equilibrated overnight at room temperature. Gelatinization properties were determined by heating the sample pan and an empty sealed pan as a reference in the differential scanning calorimeter (DSC) (Diamond DSC, PerkinElmer, Waltham, MA, USA) from 30 °C to 90 °C, at a scanning rate of 10 C/min. The transition temperatures (i.e., the onset (To), peak [7], and conclusion (Tc.) temperatures), temperature range (∆T), and the enthalpy (∆H) of gelatinization were determined from the thermograph using Pyris^TM^ software version 12 (PerkinElmer, Waltham, MA, USA). To analyze the starch retrogradation properties, DSC pans containing gelatinized starches were kept at 4 ± 1 °C for 14 days. The sample pans were then rescanned at the rate of 10 °C /min from 30 to 90 °C. The transition temperatures (i.e., the onset (To(r)), peak (Tp(r)), conclusion (Tc(r)) temperatures), temperature range (∆T(r)), and the enthalpy (ΔH(r)) of melting of the retrograded structure were determined as previously mentioned. Degree of retrogradation (R) was calculated using the following equation [9,62]:

R (%) = ΔH(r)/ΔH × 100(4)

#### 2.5.6. Starch Pasting Properties

Pasting properties of the rice starch were determined with a Rapid Visco Analyzer (RVA-4, New Port Scientific Instrument and Engineering, Warriewater, Australia) according to the AACC International Approved Method 76–21.01 [63]. Rice starch sample (2.8–3.0 g, 7.7–12.6% moisture) was mixed thoroughly with double-distilled water (25.0–25.2 g) in the RVA aluminum canister to achieve 12% (*w/v*) starch slurry. The slurry was stirred manually using a plastic paddle for 20 s before insertion into the RVA machine. Heating and cooling program was set as followed: equilibrated at 50 °C and mixed at 960 rpm for 10 s to allow complete dispersion, declined rotation speed to 160 rpm and holding at 50 °C for 50 s, heating to 95 °C within 3 min 42 s, and holding at 95 °C for 3 min 30 s. The slurry was finally cooled to 50 °C within 3 min 48 s, and then held at 50 °C for 2 min. The RVA pasting curve was retrieved, and the pasting temperature (P_Temp_), peak time (P_Time_), peak viscosity (PV), trough viscosity (TV), and final viscosity (FV) were recorded and used to calculate the breakdown (BD = PV − TV), and setback (SB = FV − TV). The viscosity was expressed in centipoise (cP) [9].

### 2.6. Statistical Analysis

The data were reported as mean ± standard error of the mean (SEM) of 3–4 biological replicates. The independent-samples *t*-test was used to evaluate the statistically significant differences (*p* ≤ 0.05) of the mean values between groups. Statistical analyses were performed using SPSS^®^ statistics v.23 software (IBM, Armonk, NY, USA) [48].

## 3. Results

### 3.1. Rice Productivity and Grain Morphology

**Figure 1:**
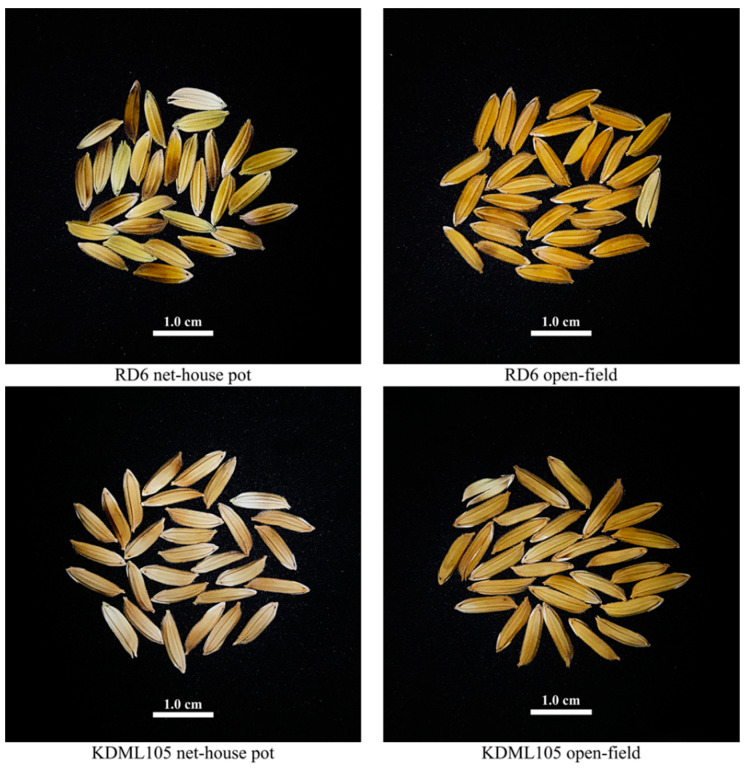
Representation of the grain appearances of two Thai elite rice cultivars grown in the net-house pot and open-field conditions.

To evaluate the effect of production systems and soil characteristics on physical characteristics of RD6 and KDML105 grains, mature rice caryopses were harvested from the net-house pot and open-field production systems. The rice productivity (i.e., panicle numbers per plant, total grain weight, 100-grain weight, and grain fertility) and grain morphology (i.e., grain length, grain width, grain length-to-width ratio, perimeter, and grain volume) of the two rice cultivars are presented in Table 2 and Table 3, respectively. Production systems and soil characteristics significantly affected the rice productivity in terms of panicle numbers per plant, total grain weight, and 100-grain weight. The RD6 and KDML105 in the open-field production system had greater panicle numbers per plant, total grain weight, and 100-grain weight than those of the same cultivars in the net-house pot production system (Table 2). However, grain fertility of both cultivars did not differ between the net-house pot and open-field conditions (Table 2). This was consistent with the physical appearance of grains at harvest, but the grain color harvested from RD6 and KDML105 cultivated in the net-house pot production system was noticeably light-colored when compared to the same cultivar planted in the open-field production system (Figure 1). Additionally, the grain length and length-to-width ratio were between 6.65 to 7.63 mm and 2.78 to 3.41 mm for RD6 and KDML105 grown in both production systems, respectively (Table 3). These ranges of the grain length and length-to-width ratio were considered as long to extra-long grains in size and medium to slender grains in shape following the International Rice Research Institute (IRRI) criteria, respectively [6]. The grain length, length-to-width ratio, perimeter, and grain volume of RD6 and KDML105 in the open-field production system were noticeably greater than those of the same cultivars grown in the net-house pot production system (Table 3). However, no statistically significant differences were found between the net-house pot and open-field production systems for grain width in both cultivars (Table 3).

### 3.2. Grain Storage Product Compositions

Production systems and soil characteristics only had a significant influence on reducing sugar content in the grains of RD6 rice cultivar (Table 4), while the effects on starch, nitrogen (N), protein, and amylose contents in the grains of the two rice cultivars were not observed (Table 4). The reducing sugar, nitrogen (N), starch, protein, and amylose contents of rice grains ranged from 0.54 mg/gFW to 0.95 mg/gFW, 719.73 mg/gFW to 748.92 mg/gFW, 1.30% to 1.41%, 7.76% to 8.37%, and 1.69% to 13.15%, respectively. These values were within the ranges reported by previous studies [9,10,19,48,49]. RD6 cultivar planted in the open-field production system contained greater reducing sugar content in the grain than that of the same cultivar cultivated in the net-house pot production system (Table 4). It is interesting that the grain storage product composition of KDML105 did not differ between the two production systems (Table 4).

### 3.3. Starch Molecular Structure

#### 3.3.1. Chain-Length Distribution of Amylopectin Branches

**Figure 2:**
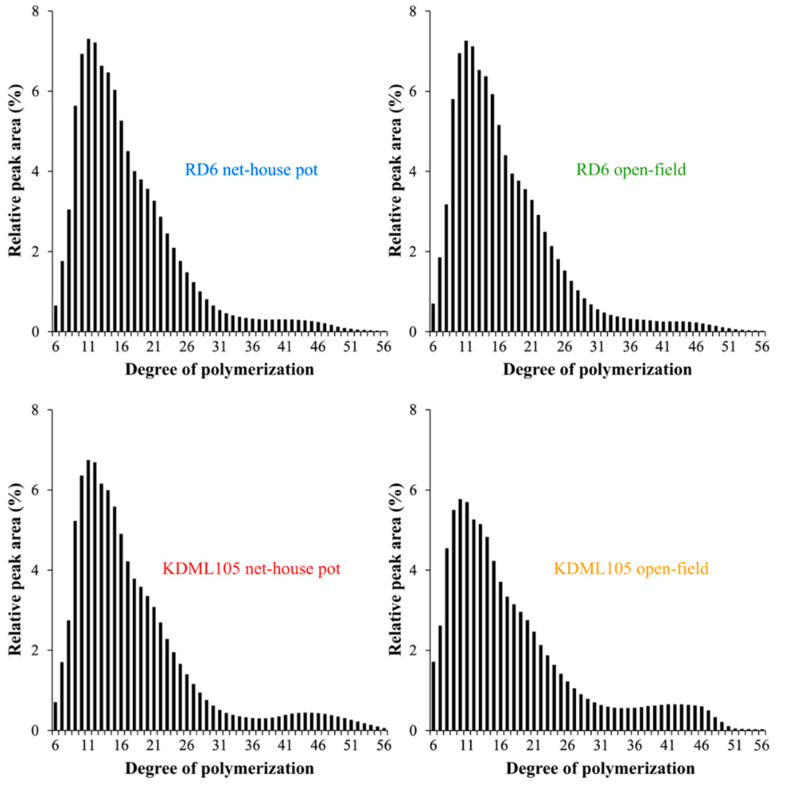
Amylopectin branch chain length distribution of grain starches from the two elite Thai rice cultivars grown in the net-house pot and open-field production systems.

The results of amylopectin fine structure of starches isolated from the two rice cultivars revealed differences in CLD of amylopectin branches between the net-house pot and open-field production systems (Table 5). Generally, amylopectin branch chains are compartmentalized into four different chain types including A chains (DP 612), B1 chains (DP 13–24), B2 chains (DP 25–36), and B3+ chains (DP ≥ 37) [9,48]. Here, the sensitivity of the HPAEC-PAD system allowed the resolve of glucans with DP ≤ 56. The amount of B2 chains separated from RD6 starch in the net-house pot production system was 9.57 ± 0.05%, and the number was significantly raised to 10.01 ± 0.07% in the open-field production system (Table 5). Despite this, significant difference in average chain length (CL) of RD6 amylopectin branch was not observed between the net-house pot and open-field production systems (Table 5). Interestingly, CL of amylopectin branches isolated from KDML105 cultivated under the net-house pot condition was 17.94 ± 0.14 AGU, and greatly raised to 19.00 ± 0.04 AGU under the open-field condition (Table 5). This is consistent with the increased proportion of B2 chains and B3+ chains (12.17 ± 0.26% and 9.38 ± 0.04%, respectively) debranched from the open-field system KDML105 starch in comparison to those (9.53 ± 0.12% and 6.80 ± 0.48%, respectively) of the net-house pot system (Table 5). In contrast, the proportion of A chains and B1 chains (30.61 ± 0.25% and 47.84 ± 0.18%, respectively) from the open-field system KDML105 starch considerably reduced from those (32.46 ± 0.33% and 51.20 ± 0.18%, respectively) of the net-house pot system (Table 5). Consistently, the chromatograms of amylopectin CLD (Figure 2) revealed the greater proportion of long B2 and B3+ chains of amylopectin branches from the open-field system KDML105 starch compared to those of the net-house pot system, while the proportion of short A and B1 chains of amylopectin branches isolated from the open-field system KDML105 starch were noticeably lower than those of the net-house pot system.

#### 3.3.2. Starch Particle Size and Granule Size Distribution

**Figure 3:**
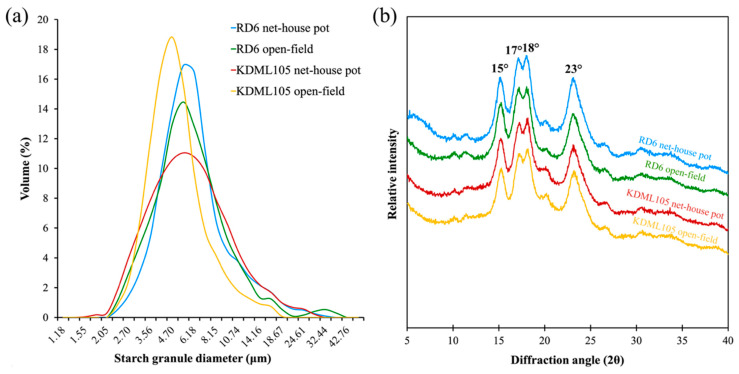
Characterization of starch from the grain of the two elite Thai rice cultivars grown in the net-house pot and open-field production systems. (**a**) granule size distribution; (**b**) X-ray diffraction patterns of starch granules.

Laser particle-size analysis was performed to verify the influence of production systems and soil characteristics on particle size and size distribution of starch granules of both RD6 and KDML105 cultivars. The results revealed significant distinction in median particle diameters (d(0.5)) and granule size distribution of KDML105 starch between the two production systems. (Table 6). According to previous study, the starch granules are categorized into three different types which are C- (<5 μm), B- (5–15 μm), and A- (>15 μm) granules [57]. Here, the KDML105 rice planted under the open-field condition exhibited a significantly higher proportion of C-type starch granules, and lower proportion of B-type and A-type starch granules than those of the same cultivar grown under the net-house pot condition (Table 6). In addition, the granule size distribution plot showed that the open-field system KDML105 starch occupied the smallest (4.70 μm) peak granule diameter, whereas, the highest (5.40 μm) peak granule diameter was dominated by the net-house pot system KDML105 starch, and the RD6 starches from both conditions (Figure 3a). Accordingly, significantly smaller median particle diameters (d(0.5)) was only observed in the open-field grown KDML105 rice starch compared to the net-house pot system KDML105 starch (Table 6). In addition, the median particle diameters (d(0.5)) of starches in the four rice grain samples (ranged from 4.73 μm to 5.75 μm) were within the range of rice starch particles [19].

#### 3.3.3. Crystalline Structure and Relative Crystallinity

The impact of production systems and soil characteristics on the crystalline structure and relative crystallinity of RD6 and KDML105 starches were presented in Figure 3b and Table 6. The typical A-type diffraction pattern, comprising the strong X-ray unresolved peak at diffraction angles (2θ) of 17° and 18° and two single peaks at 15° and 23°, was observed in starch granules separated from the flours of all samples (Figure 3b). Additionally, the weak X-ray reflection peaks of amylose–lipid complexes appeared at diffraction angles (2θ) of 20.16° in this study (Figure 3b) [57,64]. Interestingly, the intensity of the amylose–lipid peak of the net-house pot system RD6 starch was distinctly higher than that of the open-field system RD6 starch (Figure 3b). In addition, significant differences in the relative crystallinity of RD6 starch granules were detected between the two different production systems (Table 6). The A-type crystalline polymorph of RD6 starch in the open-field condition had lower relative crystallinity than that of the net-house pot production (Figure 3b and Table 6). The relative crystallinity ranged from 33.52% to 38.01% for all the two tested rice cultivars (Table 6), which is consistent with the results of the previous studies [10,62,65].

### 3.4. Starch Physicochemical Properties 

#### 3.4.1. Gelatinization and Retrogradation Properties

The gelatinization and retrogradation behaviors of starches were characterized by the transition temperatures and the enthalpy of gelatinization and melting of the retrograded structure. The results revealed that the open-field system RD6 starch had a significantly lower gelatinization To, but greatly higher gelatinization Tc, ΔT, and ΔH compared to those of starch from the net-house pot system RD6 (Table 7). On the other hand, the open-field system KDML105 starch possessed an eminently higher gelatinization To, but a distinctly lower gelatinization ΔT compared to those of the net-house pot system KDML105 starch (Table 7). Additionally, data related to the melting endotherm of the retrograded starch were presented in Table 8. All parameters including transition temperatures as well as degree of retrogradation (R) of RD6 starch in the net-house pot production system were comparably greater than those of the open-field production system (Table 8). On the other hand, the retrogradation To(r), Tp(r), and Tc(r) of KDML105 starch grown under the net-house pot condition at were significantly lower than those of the open-field condition starch (Table 8). Nevertheless, no differences in ΔT(r), ΔH(r), and the percentage of retrogradation (R%) of KDML105 starch gel were observed between the two growing conditions (Table 8).

#### 3.4.2. Starch Swelling Power and Water Solubility

The influence of production systems and soil characteristics on starch swelling power and solubility of RD6 and KDML105 rice cultivars were shown in Table 9. The swelling power and water solubility of starches of all rice grain samples were in the range of 23.68% to 39.33% and 3.97% to 13.17%, respectively. These values were consistent with several previous studies [10,19,62]. No statistically significant differences in the swelling power and water solubility of starches isolated from both cultivars were observed between the two different production systems (Table 9). Nonetheless, the swelling power (23.68 ± 0.52 g/g) of the open-field system KDML105 starch tended to be lower than that (25.21 ± 0.49 g/g) of the net-house pot system KDML105 starch (*p* = 0.075) (Table 9).

#### 3.4.3. Pasting Properties

The pasting behavior of starches from RD6 and KDML105 rice grown under the two different production systems are summarized in Table 9 and Figure S2. Despite changes in starch molecular structure, production system did not have an influence on most pasting characteristics, including P_Temp_, P_Time_, TV, BD, FV, and SB of RD6 and KDML105 starches (Table 9 and Figure S2). However, the KDML105 rice cultivar grown under the open-field production system possessed lower PV of 2960.50 ± 97.41 cP than that of the same cultivar planted under the net-house pot production system with the PV of 3279.00 ± 67.22 cP (Table 9 and Figure S2).

## 4. Discussion

Besides the genetic characteristics of rice cultivars, production systems, soil physical and chemical characteristics (e.g., soil porosity, respiration, moisture, and nutrients), and climatic conditions play significant roles in controlling its yield and grain quality [26,66,67,68]. The rice yield components (i.e., panicle numbers per plant, total grain weight, 100-grain weight, and grain fertility) and grain quality (i.e., physical characteristics, chemical compositions, and starch physicochemical properties), in turn, are the principal parameters determining the price of rice in the market and ultimately the income of farmers [69]. Furthermore, the quality parameters of rice grain and rice starch are crucial for its use as raw materials for carbohydrate-rich food production and other rice processing industry [16,70,71]. Here, the results regarding which the two distinct growth conditions with differing production systems and soil characteristics directly or indirectly affect yield, grain quality, and starch physicochemical properties of the two elite Thai rice cultivars are discussed. 

### 4.1. Effect of Production System and Soil Characteristic on Yield and Grain Appearance Quality of Rice

The presence of a large proportion of sand in cultivation soil is accompanied by a small amount of OM, which results in low available water capacity (AWC), cations exchange capacity (CEC), and macronutrient and micronutrient preservation of sandy soil. This typically leads to the diminutions of rice yield (such as spikelet number, panicle number, and grain weight) [29,30,72]. Nonetheless, our findings revealed that the open-field condition with a higher proportion of sand, and lower OM, NPK contents and CEC in the soil rendered greater panicle numbers, total grain weight, and 100-grain weight of RD6 and KDML105 when compared to the same cultivar planted under the net-house pot condition with excessively higher OM, CEC, and especially NPK contents in the potting soil mixture (Table 1 and Table 2). This phenomenon was also previously observed [36,73]. The excessive N and K application in the net-house pot production system might inhibit Fe uptake into the root cell of rice [34]. Overabundant N could affect the mobility of Fe in soil, while excess K increased the root oxidizing power of rice, together resulting in oxidation of Fe^2+^ to Fe^3+^, which prevented it from being taken up by the rice root [34]. Fe-deficiency in turn could result in chlorophyll shortage in photosynthetic tissues, reduced photosynthesis, decreased biomass production, which eventually hinder plant development and yield [35]. Fe also plays an important role in a number of plant cellular processes, and acts as a cofactor of several enzymes necessary for a wide range of biological functions [35]. In addition to the existence of soil nutrient, other factors such as light intensity within rice cultivation location could influence yield and grain quality of rice [74,75,76], by which the traditional open-field system provided higher light intensity than the inside of net-house system [76]. Consequently, rice plants grown in the open-field production system possessed higher net photosynthetic and electron transport rates and photochemical efficiency than those cultivated in the net-house pot production system [77]. The elevated photosynthate production in rice leaves is accompanied by an increment in its transport to the grain filling site for biosynthesis and accumulation of starch components (i.e., amylose and amylopectin) in the rice grains [74,75], allowing for higher total grain weight and 100-grain weight than the rice plants cultivated under the net-house pot production system [74,75]. Additionally, panicle number and grain weight are particularly important components of grain yield and quality [49,78,79,80]. The observed greater panicle number and grain weight of RD6 and KDML105 from the open-field cultivation would serve higher agronomic yields and better grain quality to rice farmers [69,81,82]. Although rice grain weight typically had positive correlation with the percentage of grain fertility [83], such correlation was not observed in both rice cultivars under this study conditions. The percentage of grain fertility of RD6 and KDML105 cultivated under the open-field condition did not vary from the net-house pot production (Table 2). Therefore, it is suggested that the higher total grain weight and 100-grain weight of RD6 and KDML105 cultivated under the open-field condition may result from the larger grain size (i.e., grain length) in this study (Table 3). Interestingly, significant increases in grain width, grain length (grain size), and grain length-to-width ratio (grain shape) of rice grown in soil containing low N and K contents were observed in previous works [31,37]. This agrees with our findings, which showed that physical quality of milled RD6 and KDML rice grains including grain length and length-to-width ratio increased tremendously when grown under the open-field condition with lower N and K contents in soil (Table 1 and Table 3).

### 4.2. Effect of Production System and Soil Characteristic on Grain Storage Product Compositions

In addition to external appearance (i.e., grain size and grain shape), production system and soil physical and chemical characteristics also considerably affected the grain internal quality (i.e., reducing sugar and starch contents, crude protein content, and the amylose to amylopectin ratio), ultimately influencing eating and cooking quality of cooked rice grains [24,26,84,85]. Water-soluble carbohydrates (such as total reducing sugars) provide precursors (i.e., ADP glucose) for starch biosynthesis in the developing rice grain; therefore, changes in total reducing sugars leads to alteration of starch accumulation in the developing rice grain [86]. Sand-loam soil containing low NPK and OM contents could promote the activities of starch biosynthesis enzymes including ADP-glucose pyrophosphorylase, soluble starch synthase, and starch branching enzyme in the developing cereal grains, allowing for the increment of amylose, amylopectin, and total starch accumulating rate in the developing cereal grains [87]. On the other hand, the findings of Singh et al. [64] demonstrated a decline of amylose accumulation in the rice grains caused by an increase of N application to the soil. However, in the current study, we only observed significant increment in reducing sugar content in the grains of RD6 grown under the open-field sandy soil, while the difference in grain starch content was not seen when compared to the RD6 grown in the net-house potting soil mixture with larger amounts of NPK and OM (Table 1 and Table 4). The application of N fertilizer at a high level could enhance the rice-protein and nutritional quality by increasing N concentration and storage protein accumulation in rice endosperm [24,64,88]. The rice plant evolved numerous mechanisms, including modification of nutrient acquisition programs and detoxification pathways with the incorporation of N into important molecules, such as amino acids, proteins, and nucleic acids to enable functional flexibility under the stress without affecting cellular and developmental and physiological processes [35]. However, our present findings did not find significant differences in nitrogen (N), storage protein, and amylose contents in the grains of RD6 and KDML105 between the net-house pot and open-field production systems despite the differences in soil N and other soil characteristics (Table 1 and Table 4). Collectively, soil physical and chemical characteristics did not have statistically significant effects on the accumulation of major storage product composition including starch, N, protein, and amylose in RD6 and KDML105 grains (Table 4). This might be explained by the other environmental factors, such as cultivating location, climatic conditions, daylight period, and temperature, which could act in concert to influence storage biomolecule compositions in the rice grains [22,24,26,89]. Furthermore, growth temperatures during the grain filling stages also had extreme effects on protein and amylose accumulations. The accumulation of crude protein and amylose contents in the rice grains obviously increased when rice was grown at high and low temperatures, respectively [89,90,91]. The glucan chains of the grain linear amylose are synthesized through the action of granule-bound starch synthase I (GBSSI) activity [92], whose enzyme activity increases when the rice plant is grown under low environmental temperature of 18 °C [93]. This could be due to the enhanced expression of *wx* gene encoding GBSSI under the low temperature [93]. However, our results exhibited small variation in the environmental temperatures and humidity patterns during the grain filling stages between the two different cultivation locations (Figure S1). The average of growth temperatures in the net-house pot and open-field production systems were 29.20 °C and 28.73 °C, respectively, while the average of growth humidity in the net-house pot and open-field production systems were 60.78% and 72.32%, respectively (Figure S1). As a result, significant differences in nitrogen, crude protein, and amylose contents in the grain of the two rice cultivars were not found between the two production systems and soil characteristics in this study (Table 4). Hence, the temperature and humidity might present a greater contribution to storage compound accumulation than soil characteristics in this study.

### 4.3. Soil Characteristic Effect on Amylopectin Fine Structure

The results of amylopectin CLD separated from the two rice starches in the net-house pot and open-field production systems showed that proportion of short A chains was in the range of 30.61% to 34.01%, which was evidently higher than their proportions of B3+ long chains (3.75–9.38%) (Table 5). With the short A chain and B3+ long chain ratio, RD6 and KDML105 starches were considered A-type starches, which is usually observed in rice and other cereal starches (e.g., wheat, common corn, barley) [94]. Little information on the influence of production system and soil characteristics on the structural characteristics of amylopectin has been acquired before. Here, we demonstrated substantial difference in the amylopectin fine structure of RD6 and particularly KDML105 rice cultivars between the two production systems with distinct soil chemical characteristics (Figure 2 and Table 5). Under the open-field production system with a lower soil nutrient content, only the proportions of B2 long chains (DP 25–36) of RD6 amylopectin significantly increased compared to that of the same cultivar grown under the net-house production system (Table 1 and Table 5). For KDML105 amylopectin, the proportions of short A and B1 chains (DP 6–12 and DP 13–24) greatly declined, while its B2 and B3+ long chains (DP 25–36 and DP ≥ 37) and the average chain length (CL) significantly increased under the open-field production system with lower soil nutrient contents (Table 1 and Table 5). Consistently, it was previously shown that starches from two *indica* rice cultivars grown in low N soil had small amount of amylopectin short branch chains and large amount of amylopectin long branch chains, resulting in high CL [95].

### 4.4. Compositional Influences of Starch on Its Median Particle Size, Granule Size Distribution, and Relative Crystallinity under Different Production Systems and Soil Qualities

The open-field system KDML105 rice cultivar produced higher proportion of C-type starch granules in volume frequency percentage, lower proportion of B- and A-type starch granules, and a smaller median particle size of starch (Table 5). It was previously reported that the declined amylose accumulation in the storage organs of crops was directly responsible for the reduction of its starch granule size [96,97]. However, amylose accumulation in the endosperm of KDML105 rice cultivar was not distinct between the two production systems in this study (Table 4). In addition to the amylose content, the activities of granule-bound starch synthase (GBSS) together with starch branching enzyme (SBE) play a crucial role in determining median particle size and size distribution [98,99,100]. The activities of these two starch biosynthesis enzymes were extremely affected by the soil physical and chemical characteristics [98,99,100]. Lack of their activities could prevent starch granules from reaching their normal sizes [98,99,100]. Collectively, significant differences in median particle size (d(0.5)) and size distribution between the net-house pot and open-field production systems of KDML105 rice starch might be putatively due to the changes in activities of GBSS and SBE in the endosperm of KDML105. The differential responses of the two enzymes in the endosperm of KDML105 to different growing conditions, such as production system, soil quality, and climatic environment, may contribute to genotypic adaptation and unique grain qualities of rice [22,71,94,101]. A significant effect of growing conditions with differing production systems and soil characteristics on the relative crystallinity of rice starch granules was also detected in this study (Table 5). To the best of our knowledge, the impact of production system and soil characteristic on the relative crystallinity of rice starch granules has not been examined before. The current study demonstrated a lower relative crystallinity of starch granule in the grains of RD6 planted under the open-field condition compared to that of the pot production system (Table 6). Relative crystallinity of rice starch could be largely determined by proportions of amylose and short and long chain of amylopectin branches [64,102]. The decline of starch relative crystallinity was directly affected by the increment of amylose content in rice starches [1,9,19,62,103]. However, difference in amylose content in the grains of RD6 cultivar was not found between the net-house pot and open-field conditions in this study (Table 4). Therefore, it is suggested that the relatively lower starch granule relative crystallinity of RD6 rice in the open-field production system could be due to the significant increment in percentages of B2 long-chain amylopectin branches [103,104]. On the contrary, no noticeable distinction in relative crystallinity of KDML105 starch granules was seen between the net-house pot and open-field production systems, despite its increment in the proportions of B2 and B3+ long chains under the open-field production system (Table 5 and Table 6). This presented the discrepancies in starch trait adaptation between a waxy genotype cv. RD6 and a low-amylose genotype cv. KDML105 [9,22,95], which may differentially respond to the climatic and soil conditions during grain development [105].

### 4.5. Relationship between the Compositional and Structural Features and Physicochemical Properties of Starches from the Two Rice Cultivars Grown under Varying Production Systems and Soil Characteristics

To investigate whether production system and soil characteristics affect the physicochemical properties of starches from RD6 and KDML105 rice cultivars, their endosperm starches were analyzed for swelling power, solubility, and thermal and pasting properties. Swelling power and water solubility of rice starches display the existence of interactions between water molecules and starch chains in amorphous and crystalline domains [1,19,60]. The starch swelling power is mainly promoted and inhibited by amylopectin and amylose, respectively, while its solubility is primarily influenced by the amylose content [1,19,60]. The highly water soluble starch is typically accompanied by a large quantity of amylose [1,19]. Accordingly, the water solubility of RD6 and KDML105 starches in the net-house pot production system did not vary from the water solubility of both cultivars in the open-field production as their amylose content were not different between two production systems (Table 4 and Table 9). In addition, rice starch containing a great proportion of amylopectin long branch chains and amylose would show low swelling capacity [1,19]. Nevertheless, we found that the net-house pot and the open-field grown RD6 starches equally swelled despite a different in their long amylopectin B2 chains (Table 5 and Table 9). Similarly, the KDML105 starches from both production systems also swelled to the same extent despite the varying amylopectin long B2 and B3+ chains proportions (Table 5 and Table 9). Overall, although production system and soil characteristic contributed to the distinctions in CLD of amylopectin branches of both genotypes between the net-house pot and open-field production systems (Table 5), they did not render the difference in their swelling power (Table 5 and Table 9).

Interestingly, the difference in the starch gelatinization of RD6 and KDML105 between the two production systems were detected from differential scanning calorimetry (DSC), which is the thermal method for evaluating the gelatinization of starch [106]. The distinctions in the onset of gelatinization of RD6 and KDML105 starches were evident between the two production systems (Table 7). The open-field system RD6 starch displayed a lower To compared to the net-house pot system RD6 starch (Table 7). Conversely, the open-field system KDML105 starch provided a much higher To than the net-house pot system KDML105 starch (Table 7). The presence of a small amount of short A chains and a large amount of long B3+ chains within starch granules could be associated with the stronger ordered structure of amylopectin double helices, including crystalline structure, which required a higher temperature to initiate their dissociation and eventually resulted in high gelatinization To [1]. Additionally, the large proportion of C-type granules and a small median particle size of rice starch also elevated the starch gelatinization temperature [48]. Accordingly, the open-field system KDML105 starch, consisting of a greater proportion of small C-type granules and a smaller median granule size, displayed higher gelatinization To in comparison to the net-house pot system KDML105 starch (Table 6 and Table 7). The significant increment in the conclusion temperature (Tc), temperature range (∆T), and the enthalpy change (∆H) of gelatinization of the lower crystallized RD6 starch may be influenced by an increase in the amount of B2 long chains (DP 25–36) under the open-field production system (Table 5, Table 6 and Table 7) [107]. The presence of a large proportion of B2 chains of amylopectin branches in the open-field system RD6 starch (Table 5) could contribute to the stabilization of the molecular ordered double helices structures within the granules [108]. In addition to the melting of the crystalline structures, distribution of starch granules with greater strength of the ordered structure in the population [96] and greater heterogeneity of the granule structure [109,110] could require greater thermal energy to disrupt the ordered structure in the starch granule population, resulting in an increase in gelatinization temperatures and the enthalpy change (∆H) of starch gelatinization. 

The retrogradation properties of the starch gels upon storage at 4 °C for 14 days have been shown to associate with amylose content, the CLD and CL of amylopectin branches [48,61,62,107,111]. Starch with a large amount of amylose and long amylopectin branch chains tends to form double helices, gel network, and ultimately strong starch gel upon reassociation under refrigerated storage conditions, which contribute to an increment in the melting temperature and enthalpy of the retrograded structure, and thus degree of retrogradation of starch [48,62,107,111]. Interestingly, the open-field system RD6 retrograded starch consisted of a larger amount of B2 long chains of amylopectin branches (Table 5). However, all retrogradation parameters of the open-field system RD6 retrograded starch diminished considerably when compared to those of the net-house pot system (Table 8). For low-amylose, non-waxy KDML105 rice starch, considerable increases in transition retrogradation temperatures (i.e., To(r), Tp(r), and Tc(r)) could be attributed to high amounts of amylopectin B2 chains and B3+ chains under the open-field production system (Table 5 and Table 8) [48,62,107,111].

In addition, rice starch which comprises low proportion of short amylopectin A chains but a high proportion of B3+ chains, displayed high pasting temperature (P_temp_), peak viscosity (PV), and final viscosity (FV) but low breakdown (BD) values [19,112]. This is due to strong interaction with paralleled packing of long double helices to sustain the structural integrity of the swollen granule [9,19,112]. In the current study, the open-field system RD6 starch with a higher proportion of B2 chains in amylopectin showed similar pasting behaviors to the net-house system RD6 starch (Table 5 and Table 8). However, the open-field system KDML105 starch with a smaller proportion of amylopectin short A chains and a higher proportion of B3+ chains displayed a lower peak viscosity (PV) compared to the net-house pot system KDML105 starch (Table 5 and Table 8). This could be attributed to its larger proportion of C-type granules and a smaller median granule size, resulting in smaller swollen granules upon heating and shearing compared to the net-house pot system KDML105 starch (Table 6). These small swollen granules are more susceptible to thermal and mechanical shear than the large swollen granules [9,19]. They become easily and rapidly disrupted under heating and shearing force condition, leading to a lower PV of the open-field system KDML105 starch paste [9,19].

## 5. Conclusions

The yield, grain quality, and starch compositional, structural, and physicochemical characteristics of the two elite Thai rice cultivars were significantly influenced by the two different production systems with differing soil qualities. The presence of nutrients, especially nitrogen (N), in the soil was likely responsible for significant differences in yield, grain morphology, and starch characteristics of RD6 and KDML105 between the two production systems. The influences of the soil’s chemical characteristics on the compositional and structural features of the open-field system RD6 starch were also evident. Considerable increases were found in the gelatinization Tc, ΔT, ΔH values, therefore, the cooking of RD6 grains harvested from the open-field production system may require more time and energy. However, it could provide a similar texture to the pot-grown RD6 starch upon cooking due to its similarity in SB viscosity. Such properties could directly affect demand, acceptability, and repeat-purchasing decision of rice consumers. Moreover, the cultivation of KDML105 rice under lower soil nutrient contents in the open-field production system could substantially enhance its grain yield components, while affecting the amylopectin and starch granule molecular structure. The changes in physicochemical features of the open-field system KDML105 starch were observed in gelatinization, retrogradation, and pasting properties. However, despite considerable differences in the retrogradation transition temperatures, KDML105 grains from both systems might provide a similar sensory texture upon cooking since the SB viscosity was not distinct between the two conditions. The similar starch gel ΔH(r) and R% also suggested comparable shelf-life quality of the starchy rice foods produced from both production systems. Overall, we showed that rice grain compositional and starch behavioral responses to the soil characteristics and production systems are complex and genotypically dependent in this study.

## Figures and Tables

**Table 1:** Physical and chemical characterization of the soil collected from three different sources.

Soil Sources	Physical Characteristics				Chemical Characteristics							
	Texture (%)			Soil Textural Classes	OM (%)	LR (t CaCO_3_ ha^−1^)	Total N (%)	Available P (mg kg^−1^)	Available K (mg kg^−1^)	CEC (cmol(+) kg^−1^)	Water pH (1:1)	EC (1:5) (dS/m)
	Sand	Silt	Clay									
Potting mix	-	-	-	-	9.56	-	0.249	1500.00	2941.30	25.60	6.28	1.834
RD6 field	86.59	11.24	2.17	Sand	0.33	1.512	0.025	8.17	9.50	3.40	5.46	0.018
KDML105 field	58.00	23.89	18.11	Sandy loam	0.25	7.600	0.032	2.67	29.23	8.20	5.41	0.006

Abbreviations: OM = organic matter; LR = lime requirement in terms of pure CaCO_3_; CEC = cations exchange capacity; and EC = electrical conductivity. Notes: The symbol “-” denotes that the data are unavailable.

**Table 2:** Productivity of the two elite Thai rice cultivars grown in the net-house pot and open-field production systems.

Cultivars	Production Systems	Panicle Number	Total Grain Weight (g)	100-Grain Weight (g)	%Fertility
RD6	Net-house pot	5.11 ± 0.35	15.32 ± 1.68	2.23 ± 0.04	91.78 ± 1.57
	Open-field	**14.67 ± 0.67** **	43.76 ± 1.25 **	2.53 ± 0.05 **	91.56 ± 1.58
KDML105	Net-house pot	4.89 ± 0.26	15.90 ± 0.77	2.34 ± 0.03	93.44 ± 1.17
	Open-field	9.33 ± 0.69 **	32.17 ± 1.16 **	2.58 ± 0.03 **	91.78 ± 1.39

Values are means ± SEM. Means in bold within each column with the ** symbol denote statistically significant difference between the two production systems at *p* ≤ 0.01 by Student’s *t*-test (*n* = 9).

**Table 3:** Grain morphology of the two elite Thai rice cultivars grown in the net-house pot and open-field production systems.

Cultivars	Production Systems	Length (mm)	Width (mm)	Length-to- Width Ratio	Perimeter (mm)	Volume (mm^3^)
RD6	Net-house pot	6.65 ± 0.03	2.39 ± 0.01	2.78 ± 0.01	14.19 ± 0.05	19.86 ± 0.20
	Open-field	7.02 ± 0.03 **	2.39 ± 0.01	2.95 ± 0.02 **	14.77 ± 0.05 **	20.84 ± 0.23 **
KDML105	Net-house pot	7.19 ± 0.09	2.22 ± 0.01	3.24 ± 0.04	14.77 ± 0.16	18.56 ± 0.34
	Open-field	7.63 ± 0.01 **	2.24 ± 0.01	3.41 ± 0.02 **	15.51 ± 0.03 **	20.13 ± 0.23 **

Values are means ± SEM. Means in bold within each column with the ** symbol denote statistically significant difference between the two production systems at *p* ≤ 0.01 by Student’s *t*-test (*n* = 9). Ten brown rice grains were randomly studied for each biological replicate.

**Table 4:** Chemical composition in the grain of the two elite Thai rice cultivars grown in the net-house pot and open-field production systems.

Cultivars	Production Systems	Reducing Sugar Content (mg gFW^−1^)	Starch Content (mg gFW^−1^)	N Content (%)	Protein Content (N × 5.95) (%)	Amylose Content (%)	Moisture Content (%)
RD6	Net-house pot	0.54 ± 0.02	719.73 ± 19.40	1.41 ± 0.06	8.37 ± 0.33	2.15 ± 0.22	9.35 ± 0.12
	Open-field	0.63 ± 0.02 *	743.89 ± 20.60	1.30 ± 0.00	7.76 ± 0.02	1.69 ± 0.09	10.73 ± 0.95
KDML105	Net-house pot	0.81 ± 0.08	723.47 ± 7.36	1.34 ± 0.01	7.97 ± 0.07	13.15 ± 0.63	8.75 ± 0.66
	Open-field	0.95 ± 0.05	748.92 ± 10.90	1.40 ± 0.05	8.31 ± 0.28	12.05 ± 0.82	8.42 ± 0.34

Values are means ± SEM. Means in bold within each column with the * symbol denote statistically significant difference between the two production systems at *p* ≤ 0.05 by Student’s *t*-test (*n* = 4 for reducing sugar, starch, and amylose content analyses; *n* = 3 for N and protein content analyses).

**Table 5:** Chain-length distributions and average chain length of amylopectin branches of grain starch of the two elite Thai rice cultivars grown in the net-house pot and open-field production systems.

Cultivars	Production Systems	Amylopectin Branch Chain Length Distribution (%)				
		DP6–12 (A Chains)	DP13–24 (B1 Chains)	DP25–36 (B2 Chains)	DP ≥ 37 (B3+ Chains)	CL (AGU)
RD6	Net-house pot	33.66 ± 0.24	52.68 ± 0.11	9.72 ± 0.05	3.95 ± 0.18	17.00 ± 0.06
	Open-field	34.01 ± 0.14	52.23 ± 0.15	10.01 ± 0.07 *	3.75 ± 0.26	16.97 ± 0.08
KDML105	Net-house pot	32.46 ± 0.33 *	51.20 ± 0.18 **	9.53 ± 0.12	6.80 ± 0.48	17.94 ± 0.14
	Open-field	30.61 ± 0.25	47.84 ± 0.18	12.17 ± 0.26 **	9.38 ± 0.04 **	19.00 ± 0.04 **

Values are means ± SEM. Means in bold within each column with the * and ** symbols denote statistically significant difference between the two production systems at *p* ≤ 0.05 and *p* ≤ 0.01, respectively, by Student’s *t*-test (*n* = 3). Abbreviations: DP = degree of polymerization of the number of glucose units in a branch chain length of amylopectin molecule; CL = average chain length; AGU = anhydroglucose unit.

**Table 6:** Starch granule size distribution (volume %), median particle size [d(0.5)], and relative crystallinity of starch granule in the grain of the two elite Thai rice cultivars grown in the net-house pot and open-field production systems.

Cultivars	Production Systems	Starch Granule Size Distribution (%)			d(0.5) (μm)	Relative Crystallinity (%)
		C-Type <5 (μm)	B-Type 5–15 (μm)	A-Type >15 (μm)		
RD6	Net-house pot	34.44 ± 1.87	61.49 ± 2.33	4.07 ± 0.46	5.75 ± 0.42	38.01 ± 0.57 *
	Open-field	38.02 ± 0.90	58.38 ± 0.73	3.60 ± 0.25	5.61 ± 0.13	35.65 ± 0.58
KDML105	Net-house pot	40.43 ± 0.20	55.49 ± 0.34 *	4.07 ± 0.54	5.65 ± 0.04 **	33.52 ± 1.06
	Open-field	57.39 ± 3.03 *	41.79 ± 2.95	0.82 ± 0.15 **	4.73 ± 0.13	34.68 ± 0.44

Values are means ± SEM. Means in bold within each column with the * and ** symbols denote statistically significant difference between the two production systems at *p* ≤ 0.05 and *p* ≤ 0.01, respectively, by Student’s *t*-test (*n* = 3 for starch granule size distribution and median particle size analyses; *n* = 4 for relative crystallinity analysis). Abbreviations: d(0.5) = the granule size at which 50% of all the granules by volume are smaller.

**Table 7:** Gelatinization properties of starches from the two elite Thai rice cultivars grown in the net-house pot and open-field production systems.

Cultivars	Production Systems	Gelatinization Properties				
		To (°C)	Tp (°C)	Tc (°C)	∆T (°C)	∆H * (J/g)
RD6	Net-house pot	58.63 ± 0.11 **	66.82 ± 0.29	76.46 ± 0.19	17.83 ± 0.22	11.31 ± 0.13
	Open-field	55.31 ± 0.09	66.60 ± 0.20	78.29 ± 0.20 **	22.98 ± 0.18 **	15.02 ± 0.06 **
KDML105	Net-house pot	57.82 ± 0.15	67.90 ± 0.16	75.88 ± 0.23	18.06 ± 0.25 **	11.18 ± 0.09
	Open-field	59.67 ± 0.16 **	67.90 ± 0.32	75.63 ± 0.21	15.96 ± 0.33	10.74 ± 0.25

Values are means ± SEM. Means in bold within each column with the ** symbol denote statistically significant difference between the two production systems at *p* ≤ 0.01 by Student’s *t*-test (*n* = 4). Abbreviations: SP = swelling power; S = solubility; To = onset temperature; Tp = peak temperature; Tc = conclusion temperature; ∆T = temperature range (Tc–To); and ∆H = gelatinization enthalpy. * Dry weight basis.

**Table 8:** Retrogradation properties of starches from the two elite Thai rice cultivars grown in the net-house pot and open-field production systems.

Cultivars	Production Systems	Retrogradation Properties					
		To(r) (°C)	Tp(r) (°C)	Tc(r) (°C)	∆T(r) (°C)	∆H * (r) (J/g)	R (%)
RD6	Net-house pot	46.04 ± 0.05 **	62.26 ± 0.05 **	70.28 ± 0.30 **	24.24 ± 0.25 **	5.12 ± 0.13 **	45.34 ± 1.02 **
	Open-field	43.46 ± 0.21	54.52 ± 0.30	64.12 ± 0.12	20.67 ± 0.20	2.89 ± 0.07	19.22 ± 0.41
KDML105	Net-house pot	45.06 ± 0.24	54.56 ± 0.11	60.37 ± 0.25	15.31 ± 0.46	2.65 ± 0.03	23.87 ± 0.31
	Open-field	46.74 ± 0.21 **	55.23 ± 0.20 *	63.51 ± 0.36 **	16.78 ± 0.49	2.44 ± 0.11	22.71 ± 1.73

Values are means ± SEM. Means in bold within each column with the * and ** symbols denote statistically significant difference between the two production systems at *p* ≤ 0.05 and *p* ≤ 0.01, respectively, by Student’s *t*-test (*n* = 3). Abbreviations: To(r) = onset temperature of retrograded starch; Tp(r) = peak temperature of retrograded starch; Tc(r) = conclusion temperature of retrograded starch; ∆T(r) = temperature range of retrograded starch (Tc(r)—To(r)); ∆H(r) = retrogradation enthalpy; and R (%) = the percentage of retrogradation. * Dry weight basis.

**Table 9:** Swelling power, solubility, and pasting properties of starches from the two elite Thai rice cultivars grown in the net-house pot and open-field production systems.

Cultivars	Production Systems	SP	S	Pasting Properties						
		(g/g)	(%)	P_Temp_ (°C)	P_Time_ (min)	PV (cP)	TV (cP)	BD (cP)	FV (cP)	SB (cP)
RD6	Net-house pot	39.33 ± 1.19	4.23 ± 0.86	68.35 ± 0.20	3.72 ± 0.03	2833.00 ± 141.42	1772.00 ± 182.78	1136.00 ± 45.34	2004.25 ± 162.52	282.25 ± 7.16
	Open-field	38.91 ± 1.15	3.97 ± 0.39	69.16 ± 0.40	3.67 ± 0.03	2583.50 ± 233.31	1348.00 ± 204.52	1310.75 ± 55.55	1564.25 ± 188.58	266.25 ± 14.65
KDML105	Net-house pot	25.21 ± 0.49	11.52 ± 0.71	72.55 ± 0.18	5.05 ± 0.09	3279.00 ± 67.22 *	1387.75 ± 75.04	1891.25 ± 65.56	2557.50 ± 129.32	1219.75 ± 49.05
	Open-field	23.68 ± 0.52	13.17 ± 0.76	72.18 ± 0.41	5.00 ± 0.08	2960.50 ± 97.41	1221.00 ± 32.71	1714.50 ± 77.45	2410.75 ± 53.43	1189.75 ± 40.81

Values are means ± SEM. Means in bold within each column with the * symbol denote statistically significant difference between the two production systems at *p* ≤ 0.05 by Student’s *t*-test (*n* = 4). Abbreviations: P_Temp_ = pasting temperature; P_Time_ = peak time; PV = peak viscosity; TV = trough viscosity; BD = breakdown; FV = final viscosity; and SB = setback.

## Data Availability

The information displayed in this study are available on request from the first author and the corresponding author.

## Supplementary Materials

The following are available online at https://www.mdpi.com/article/10.3390/foods10112601/s1, Figure S1: The temperature and relative humidity in the net-house pot and open-field production systems from rice transplanting to harvest. Figure S2: RVA pasting curves of starch from two elite Thai rice cultivars grown in the net-house pot and open-field production systems.
