# Supplementary material for: Yield, Grain Quality, and Starch Physicochemical Properties of 2 Elite Thai Rice Cultivars Grown under Varying Production Systems and Soil Characteristics
Source: Foods. 2021 Oct 27;10(11):2601. doi: 10.3390/foods10112601 (PMC8620510; doi:10.3390/foods10112601)

Supplementary Material

Figure S1. The temperature and relative humidity in the net-house pot and open-field production systems from rice transplanting to harvest.

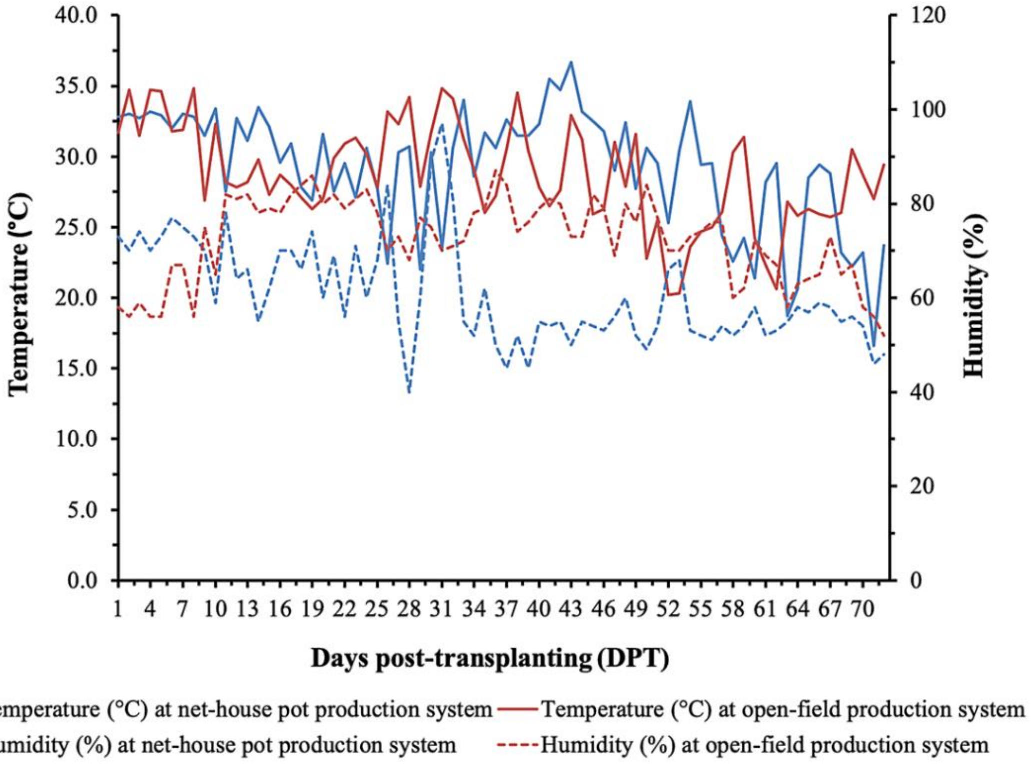

Figure S2. RVA pasting curves of starch from two elite Thai rice cultivars grown in the net-house pot and open-field production systems.

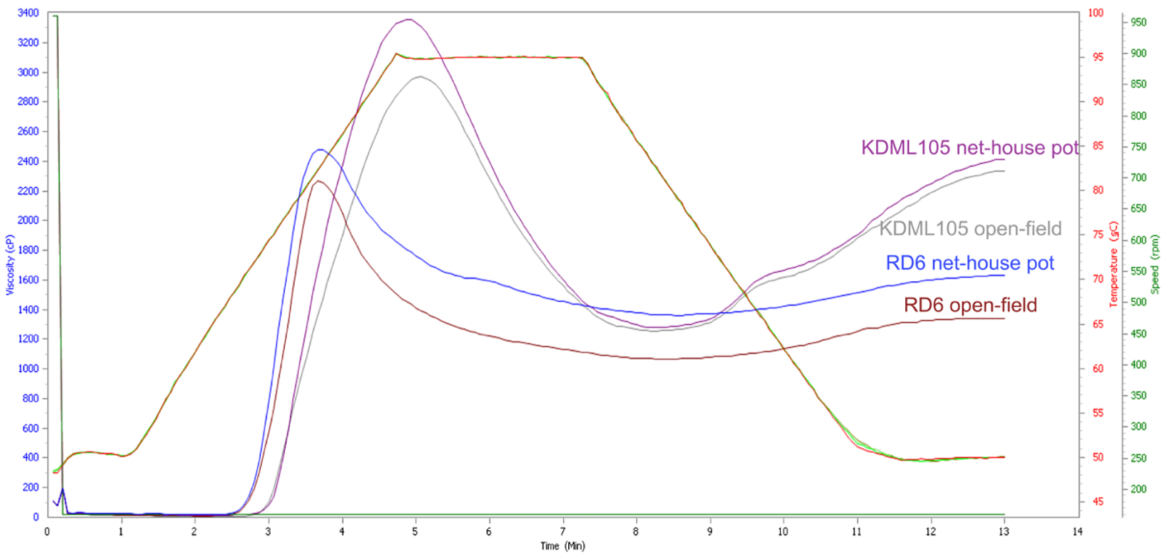

Supplement: Supplementary file 1 [file foods-10-02601-s001.zip › foods-1407863-supplementary.pdf]
